# Leveraging Cell Migration Dynamics to Discriminate Between Senescent and Presenescent Human Mesenchymal Stem Cells

**DOI:** 10.1007/s12195-024-00807-0

**Published:** 2024-07-20

**Authors:** Farshad Amiri, Panagiotis Mistriotis

**Affiliations:** Department of Chemical Engineering, Auburn University, Auburn, AL USA

**Keywords:** Stem cell migration, Senescence, Confinement, Fluid forces, Aging

## Abstract

**Purpose:**

The suboptimal clinical performance of human mesenchymal stem cells (hMSCs) has raised concerns about their therapeutic potential. One major contributing factor to this issue is the heterogeneous nature of hMSCs. Senescent cell accumulation during stem cell expansion is a key driver of MSC heterogeneity. Current methodologies to eradicate senescent hMSCs have either shown limited success or lack clinical relevance. This study leverages the inherent capacity of hMSCs to migrate toward damaged tissues as a means to discern senescent from presenescent stem cells. Given the established deficiency of senescent cells to migrate through physiologically relevant environments, we hypothesized that a microfluidic device, designed to emulate key facets of in vivo cell motility, could serve as a platform for identifying presenescent cells.

**Methods:**

We employed a Y-shaped microchannel assay, which allows fine-tuning of fluid flow rates and the degree of confinement.

**Results:**

Highly migratory hMSCs detected by the device not only demonstrate increased speed, smaller size, and higher proliferative capacity but also manifest reduced DNA damage and senescence compared to non-migratory cells. Additionally, this assay detects presenescent cells in experiments with mixed early and late passage cells. The introduction of fluid flow through the device can further increase the fraction of highly motile stem cells, improving the assay's effectiveness to remove senescent hMSCs.

**Conclusions:**

Collectively, this assay facilitates the detection and isolation of a highly potent stem cell subpopulation. Given the positive correlation between the migratory potential of administered MSCs and the long-term clinical outcome, delivering homogeneous, highly motile presenescent hMSCs may benefit patient outcomes.

**Supplementary Information:**

The online version contains supplementary material available at 10.1007/s12195-024-00807-0.

## Introduction

Human Mesenchymal Stem Cells (hMSCs) show great promise as an autologous stem cell source for tissue engineering and regenerative medicine applications. hMSCs are easily accessible, clonogenic, multipotent, non-tumorigenic, anti-inflammatory, and proangiogenic, and thus have become the most common stem cell source in clinical trials [1, 2]. Although MSC treatments lead to unambiguous beneficial effects in preclinical models, their efficacy in human phase III clinical trials is still unclear [1]. The absence of a specific and unique marker [3] for the isolation of functional hMSCs leads to the clinical use of heterogeneous populations, which can limit their therapeutic potential [4, 5] and contribute to the inconsistency in clinical outcomes. The heterogeneity in MSCs is manifested in the bulk cell population due to inherent cell-to-cell differences, including morphological, transcriptomic and functional variations between individual MSCs [6, 7]. This intrapopulation diversity is partly a consequence of their in vitro propagation to large cell numbers [8], a necessary process for cellular therapies. Analysis at the single-cell level has demonstrated that the accumulation of senescent cells as a result of stem cell culture is a key driver of MSC heterogeneity [9].

Senescent MSCs display enlarged morphology [10, 11], reduced migration capacity [12], impaired proliferation and differentiation potential [10, 11, 13–15] and a unique secretory phenotype (aka Senescence-Associated Secretory Phenotype, SASP), which includes a pool of proinflammatory molecules and proteases that remodel the surrounding matrix, and increase tissue inflammation [16]. Consequently, the emergence (~at passage 5) [17], and the subsequent accumulation of senescent cells in adult MSC cultures reduce the potency of the bulk MSC population over time, restricting the maximum population doubling level to 30-40 [18, 19]. Notably, the percentage of senescent cells is significantly higher in MSCs derived from old(er) relative to young(er) donors [20, 21], which is a major concern in regenerative medicine because older patients are most likely to receive cellular therapies. It is thus urgent to develop technologies to remove senescent cells from stem cell cultures in order to (a) reduce hMSC heterogeneity and improve standardization of cell preparations, (b) discover novel signatures for highly potent, presenescent hMSCs and (c) increase the effectiveness of hMSCs for anti-aging treatments.

Current methods to remove senescent cells from stem cell populations include genetic engineering approaches that aim to rejuvenate senescent hMSCs (reprogramming [14, 22–25]), or small chemicals that eradicate senescent cells (senolytics [26]). However, genetic approaches may use viral vectors, which may lead to tumorigenicity, while senolytics only marginally eliminate senescent hMSCs and may compromise the function of healthy cells [27–29]. Herein, we leverage the inherent capacity of hMSCs to adapt to mechanically challenging environments and migrate toward damaged tissues for the purpose of separating senescent from presenescent cells. Using a microfluidic device that recapitulates key biochemical, biophysical and topographical cues of the cell microenvironment, we demonstrate that highly migratory cells upregulate the proliferation marker Ki-67, exhibit reduced DNA damage and senescence, and display unique morphological features that set them apart from non-migratory cells. In addition, we show that cell exposure to pressure-driven flow enhances the efficacy of this device in separating senescent from presenescent adipose-derived hMSCs. Overall, our study reveals a novel approach to detect and isolate a highly motile presenescent stem cell subpopulation. Given the ineffectiveness of MSCs to reach injury sites [30] and the positive correlation between the migratory potential of administered MSCs and the long-term clinical outcomes [31], we posit that the delivery of a more homogeneous, highly motile presenescent hMSC subpopulation will yield positive clinical results.

## Results

### A Microfluidic Assay to Detect Highly Migratory and Deformable Cell Subpopulations

**Fig. 1:**
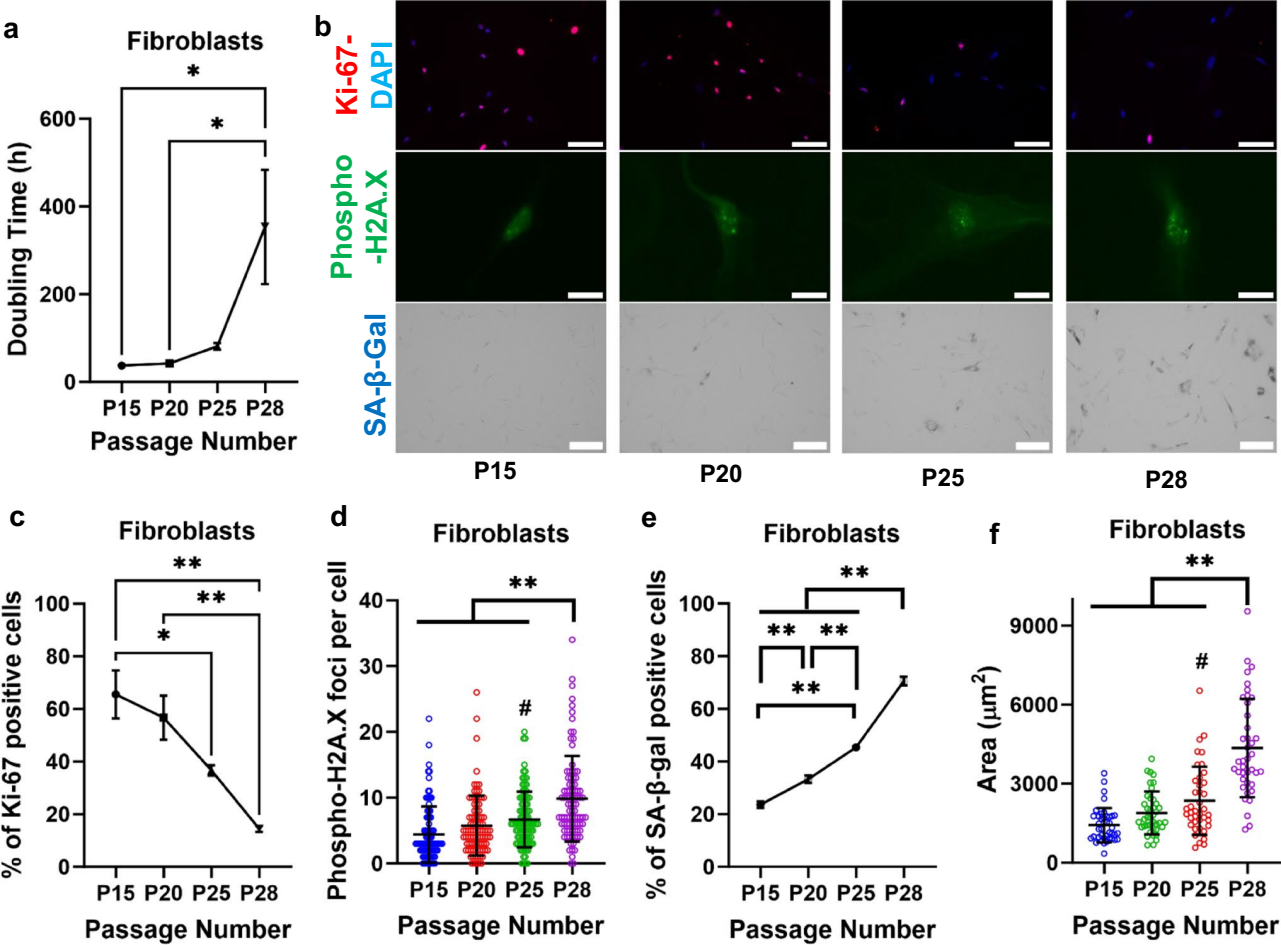
Prolonged cell culture triggers replicative senescence in human dermal fibroblasts. **a** The doubling time of fibroblasts at passages 15, 20, 25, and 28. 3 independent experiments; *p < 0.05. **b** Representative images showing levels of Ki-67 (top row), phospho-H2A.X (middle row) and SA-β-Gal (bottom row) in fibroblasts at the indicated passage (P15, P20, P25, and P28). Scale bars for Ki-67, phospho-H2A.X and SA-β-Gal are 100 μm, 20 μm and 200 μm, respectively. **c**–**e** Quantification of **b**. **c** For Ki-67 staining, at least 30 cells were analyzed per experiment; 3 independent experiments; *p < 0.05, **p < 0.01. **d** For phospho-H2A.X staining, n = 90 cells; Data pooled from 3 independent experiments; ^#^p < 0.05 relative to passage 15, **p < 0.01. **e** For SA-β-Gal staining, at least 100 cells were analyzed per experiment; 3 independent experiments; **p < 0.01. **f** Area of fibroblasts at passages 15, 20, 25, and 28. n = 40 cells; Data pooled from 2 independent experiments; **p < 0.01 and #p < 0.05 relative to passage 15. Values represent mean ± SD (**d** and **f**) or mean ± SEM (**a**, **c** and **e**).

The accumulation of senescent cells in stem cell cultures reduces the potency of the bulk cell population. Because senescent cells display reduced motility, flexibility and deformability [32–35], we hypothesized that an assay based on cell migration capacity would facilitate the segregation of presenescent and senescent cell subpopulations. To test this hypothesis, we initially employed human dermal fibroblasts, a highly migratory cell population that shares many morphological and behavioral traits with hMSCs [36]. Dermal fibroblasts also express elevated levels of the proliferation marker Ki-67 and exhibit a robust proliferation capacity for ~ 20-25 passages (Fig. 1a-c), beyond which cells undergo senescence as indicated by the increased levels of the DNA damage marker phospho-H2A.X, the upregulation of the senescence marker senescence-associated-β-galactosidase (SA-β-Gal) and the enlarged cell area (Fig. 1b, d-f). The prolonged proliferative activity of fibroblasts renders them well-suited for optimizing our assay before progressing to adult hMSCs, which lose their proliferation capacity much faster and can show signs of senescence as early as passage 5 [17].

**Fig. 2:**
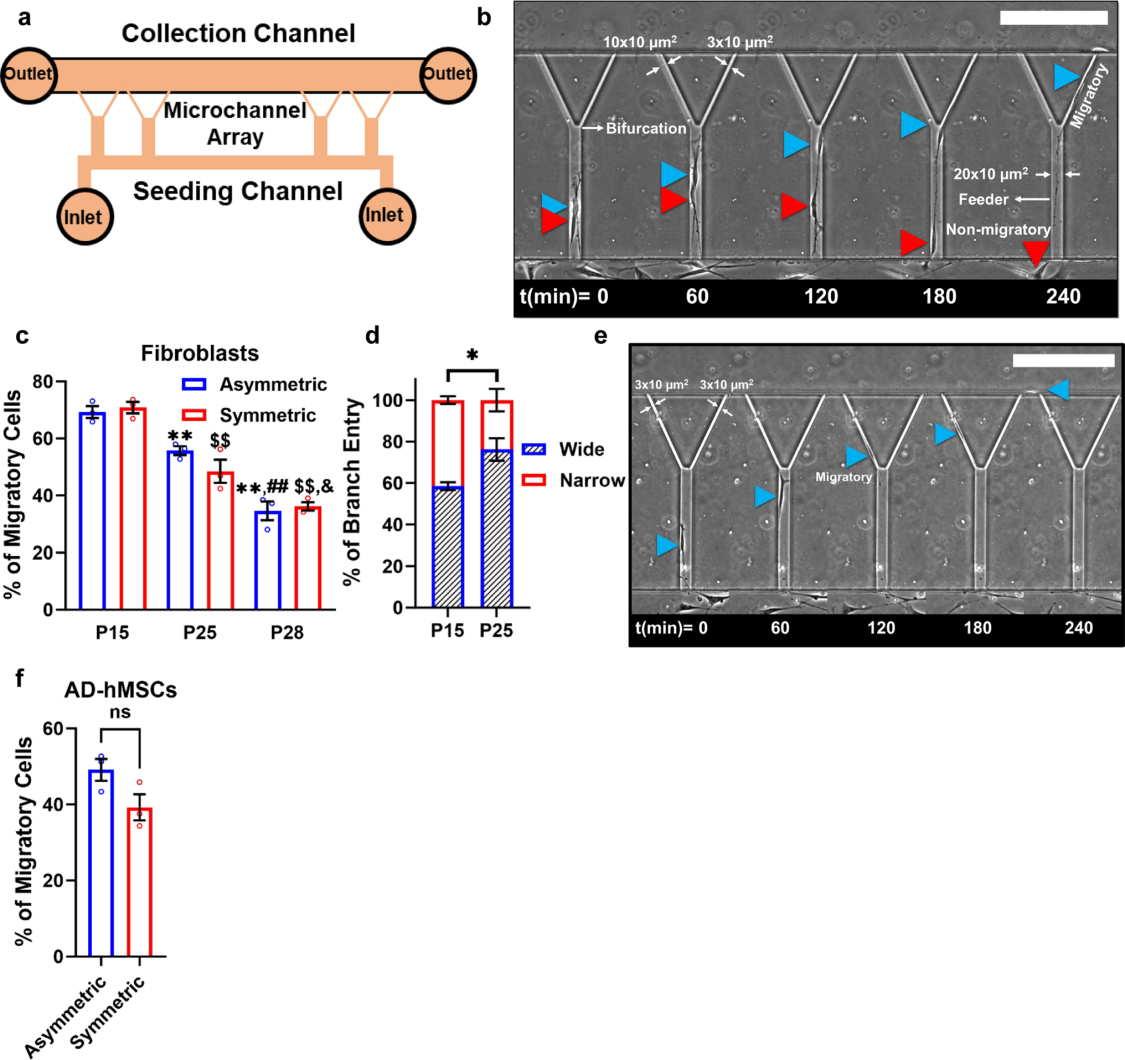
MAqCI detects highly migratory cell subpopulations. **a** Schematic representation of MAqCI. **b** Image sequence showing a migratory (blue arrowhead) and a non-migratory (red arrowhead) fibroblast in the asymmetric device. Scale bar, 200 μm. **c** Percentage of migratory fibroblasts in asymmetric or symmetric devices at passages 15, 25, and 28. At least 25 cells analyzed per experiment; 3 independent experiments; **p < 0.01 relative to passage 15 asymmetric device, ^$$^p < 0.01 relative to passage 15 symmetric device, ^##^p < 0.01 relative to passage 25 asymmetric device, and ^&^p < 0.05 relative to the passage 25 symmetric device. **d** Percentage of migratory fibroblasts at passages 15 or 25 entering the narrower or wider branch channels. At least 74 cells analyzed per experiment; 3 independent experiments; *p < 0.05. **e** Image sequence showing a migratory fibroblast (blue arrowhead) in the symmetric device. Scale bar, 200 μm **f**. Percentage of migratory adipose-derived hMSCs (AD-hMSCs) in asymmetric or symmetric devices at passage 6. At least 85 cells analyzed per experiment; 3 independent experiments. Values represent mean ± SEM.

A Microfluidic Assay for quantification of Cell Invasion (MAqCI) enables the detection and isolation of highly motile tumor cells in real-time and at the single-cell level [37, 38]. This microfluidic platform is composed of two parallel collagen type I-coated seeding and collection channels separated by a series of collagen type I-coated Y-shaped microchannels aligned in a ladder-like configuration. The Y-shaped channels consist of a larger feeder channel (Width(*W*) = 20 μm and Height(*H*) = 10 μm), and a bifurcation leading to two asymmetric, narrower branches (left branch: *W* = 10 μm and *H* = 10 μm; right branch: *W* = 3 μm and *H* = 10 μm) (Fig. 2a, b). The dimensions and geometry of the Y-shaped channels were selected to recapitulate various facets of in vivo cell motility, such as the pore sizes encountered in the interstitial tissue [39], migration inside 3D longitudinal, channel-like tracks (larger feeder channel) [40], decision-making (bifurcation), and cytoskeletal rearrangement/confined cell migration (narrower branches). Cells that entered the feeder channel, reached the bifurcation region and migrated through one of the branch channels, were defined as migratory (Fig. 2b and suppl. Video 1) [37, 38]. Non-migratory cells were those whose locomotion was restricted to the feeder channel [37, 38] (Fig. 2b and suppl. Video 2). Using this assay, we evaluated how prolonged in vitro culture influenced the migratory potential of fibroblasts. We found that the percentage of migratory cells decreased with time in culture, dropping from ~ 70% in passage 15 to ~ 35% in passage 28 (Fig. 2c). Interestingly, with increasing passages, a greater proportion of migratory cells entered the wider (10 μm) rather than the narrower (3 μm) branch channels (Fig. 2d), likely due to the larger size (Fig. 1f) and reduced flexibility and deformability of late-passage cells [32–35]. This finding prompted us to investigate whether reducing the size of the wider branch channel would hinder migration of late passage/senescent cells. Performing migration experiments in symmetrical 3/3 μm branch channels (Fig. 2e) yielded comparable percentages of highly migratory fibroblasts, as those in asymmetrical design (Fig. 2c), suggesting that highly motile cells identified by the device could deform their cytoskeleton and nucleus to enter narrow constrictions even at late passages. To further underscore the effectiveness of MAqCI in discerning migratory cells, we performed migration experiments with late passage fibroblasts (passages 25-28) using straight microchannels (*W* x* H* x* L* = 10 x 10 x 200 μm^3^). While ~ 35-50% of late passage fibroblasts could enter one of the narrower branch channels of MAqCI (Fig. 2C), ~ 80% of similar passage fibroblasts exited straight channels (Suppl. Figure 1a), indicating that straight channels were inadequate to separate migratory from non-migratory cell populations.

To test if MAqCI could detect a migratory stem cell subpopulation, we performed experiments in asymmetric and symmetric devices using passage 6 human adipose-derived MSCs. These cells exhibited senescence levels comparable to those observed in passage 25 human fibroblasts; ~ 55% of passage 6 hMSCs were stained positive for SA-β-Gal staining (suppl. Fig. 1b and Fig. 1b and e). Consistent with our data obtained using passage 25 fibroblasts, the percentage of adipose-derived hMSCs that demonstrated increased migration capacity was ~ 40-50% as shown using both designs (Fig. 2f). These data indicated that hMSCs contained a highly motile subpopulation of cells capable of invading confining environments.

### Highly Motile Cells Display Distinct Morphological and Functional Features that Distinguish them from Non-migratory Cells

**Fig. 3:**
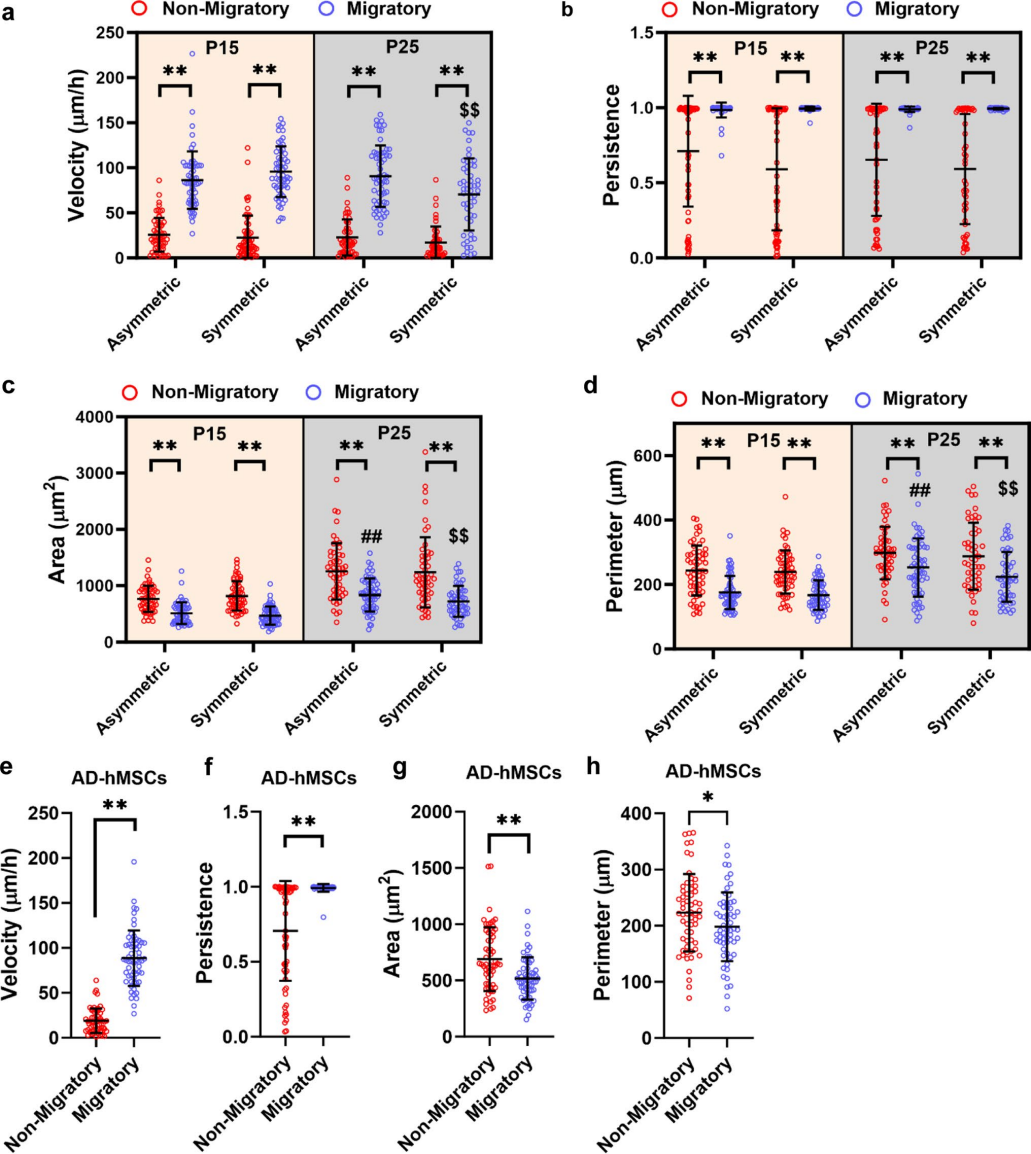
Morphological and functional comparison between migratory and non-migratory cell subpopulations **a** velocity, **b** persistence, **c** area and **d** perimeter of migratory and non-migratory fibroblasts in asymmetric or symmetric devices at passages 15 and 25. n ≥ 52 cells; Data pooled from 3 independent experiments; ** p < 0.01, ##p < 0.01 relative to passage 15 migratory in the asymmetric device, and $$p < 0.01 relative to passage 15 migratory in the symmetric device. **e** Velocity, **f** persistence, **g** area and **h** perimeter of migratory and non-migratory adipose-derived hMSCs in asymmetric or symmetric devices at passage 6. n = 60 cells; Data pooled from 3 independent experiments; *p < 0.05, ** p < 0.01. Values represent mean ± SD

Next, we performed a comprehensive analysis of phenotypic parameters to unveil distinct morphological and functional characteristics differentiating highly migratory cells from their non-migratory counterparts. To ensure a fair comparison between the two cell populations, these measurements were carried out in the feeder channel. Our findings indicated that regardless of cell passage, migratory fibroblasts had higher velocity and were more persistent than non-migratory cells (Fig. 3a, b). Additionally, migratory fibroblasts consistently displayed smaller area and perimeter compared to their non-migratory counterparts (Fig. 3c, d). This pattern persisted even in passage 25 fibroblasts, which, on average, were larger than early passage cells (Figs. 1f, 3c, d). Interestingly, the two cell populations displayed similar aspect ratios and solidity, suggesting no difference in their capacity to elongate and form protrusions (suppl. Fig. 1c and d). Our data further demonstrated that both microfluidic designs were equivalent in segregating migratory and non-migratory populations (Fig. 3a-d). Thus, subsequent experiments were carried out using one of the two designs, specifically the symmetric branch channels. Migratory adipose-derived hMSCs, similar to highly motile fibroblasts, exhibited higher velocity and persistence and had smaller area and perimeter compared to non-migratory cells (Fig. 3e-h). In sum, these findings indicate that the use of MAqCI permits the detection of a migratory cell subpopulation with specific morphological and functional traits.

### Highly Motile Cells are More Proliferative and Display Reduced Levels of DNA Damage and Senescence

**Fig. 4:**
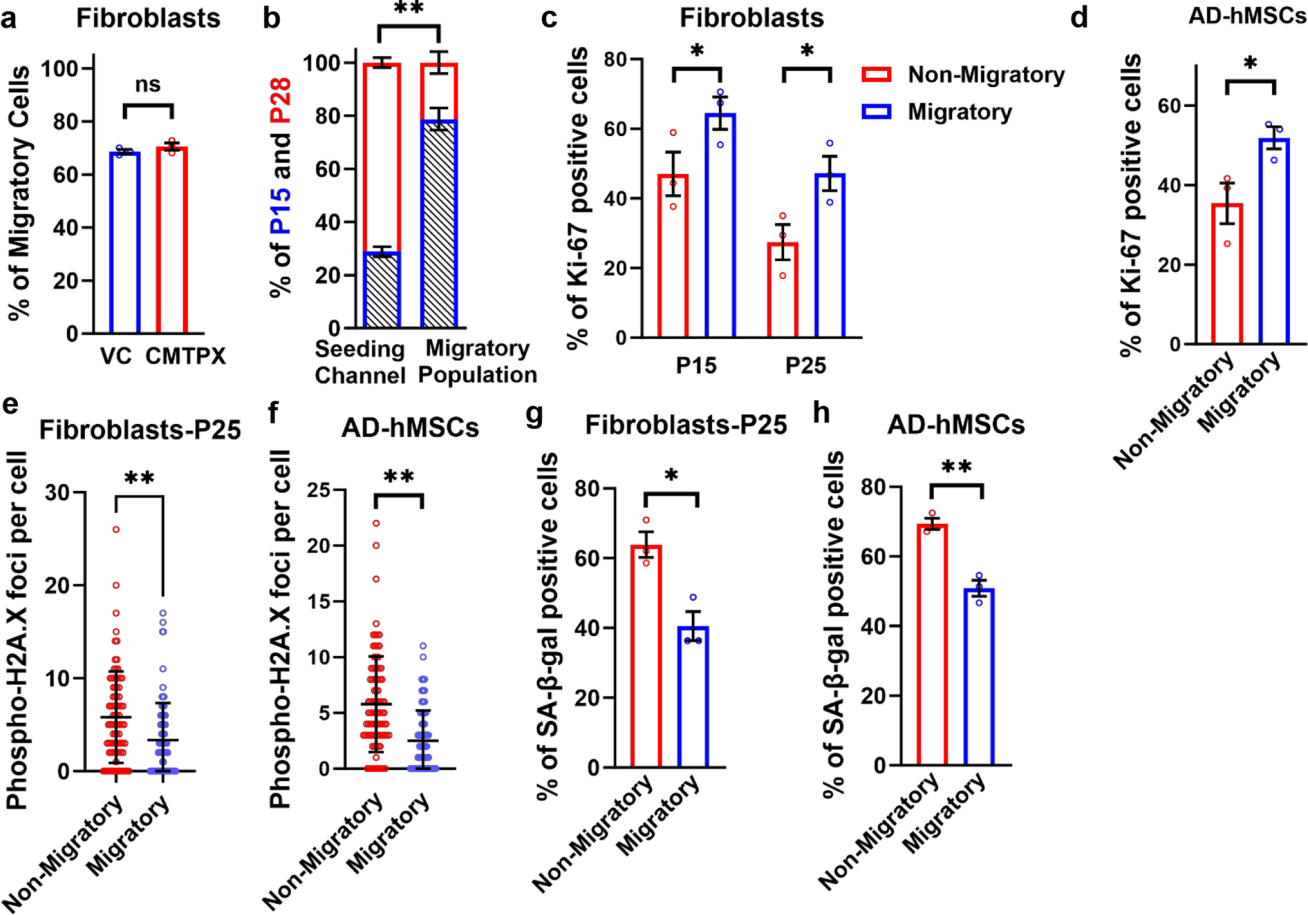
MAqCI enriches for a proliferative and presenescent cell subpopulation. **a** Percentage of migratory fibroblasts in the symmetric design at passage 15. Cells were pre-treated with vehicle control (VC) or CMTPX. At least 150 cells analyzed per experiment; 3 independent experiments. **b** Percentage of passage 15 and passage 28 fibroblasts in the seeding channel of a symmetric device and in the migratory cell population after 16 hours. At least 43 cells analyzed per experiment; 3 independent experiments; **p < 0.01. **c** Percentage of migratory and non-migratory fibroblasts stained positive for Ki-67 in the symmetric device at passages 15 and 25. At least 28 cells analyzed per experiment; 3 independent experiments; *p < 0.05. **d** Percentage of migratory and non-migratory adipose-derived hMSCs stained positive for Ki-67 in the symmetric device at passage 6. At least 37 cells analyzed per experiment; 3 independent experiments; *p < 0.05. **e** The number of phospho-H2AX foci in migratory and non-migratory fibroblasts in the symmetric device at passage 25. n = 90 cells; Data pooled from 3 independent experiments; **p < 0.01. **f** The number of phospho-H2AX foci in migratory and non-migratory adipose-derived hMSCs in the symmetric device at passage 6. n = 90 cells; Data pooled from 3 independent experiments; **p < 0.01. **g** Percentage of migratory and non-migratory fibroblasts stained positive for SA-β-Gal in the symmetric device at passage 25. At least 28 cells analyzed per experiment; 3 independent experiments; *p < 0.05. **h** Percentage of migratory and non-migratory adipose-derived hMSCs stained positive for SA-β-Gal in the symmetric device at passage 6. At least 22 cells analyzed per experiment; 3 independent experiments; **p < 0.01. Values represent mean ± SD (**e** and **f**) or mean ± SEM (**a–d**, **g** and **h**).

The observation of reduced cell area and perimeter in highly motile cells prompted us to investigate whether MAqCI could detect presenescent cells within heterogeneous populations. To this end, we pre-labeled presenescent passage 15 fibroblasts with the CellTracker CMTPX red dye, which had no impact on the fraction of highly motile cells (Fig. 4a). Next, these fluorescently labeled cells were mixed with senescent passage 28 cells at a ratio of 1:3 and introduced into the symmetric device to assess the relative abundance of each cell population cells in highly migratory cells. Although only ~ 25% of cells in the seeding channel originated from passage 15, this percentage increased to ~ 80% in the migratory cell population (Fig. 4b), suggesting that MAqCI could increase the fraction of presenescent cells. To further verify this, we quantified the proportion of highly migratory and non-migratory cells expressing Ki-67, a protein that accumulates in the nucleus of actively proliferating cells. We found that the migratory fibroblast and adipose-derived hMSC population contained a higher percentage of proliferating cells compared to non-migratory cells (Fig. 4c and d). Moreover, migratory cells exhibited reduced expression of phospho-H2A.X and SA-β-Gal (Fig. 4e-h), indicating lower levels of DNA damage and senescence, respectively. Collectively, these data demonstrate that MAqCI enriches for faster, smaller and more deformable presenescent hMSCs.

### Application of Pressure-Driven Flow Improves the Efficacy of MAqCI to Remove Senescent Adipose-Derived hMSCs

**Fig. 5:**
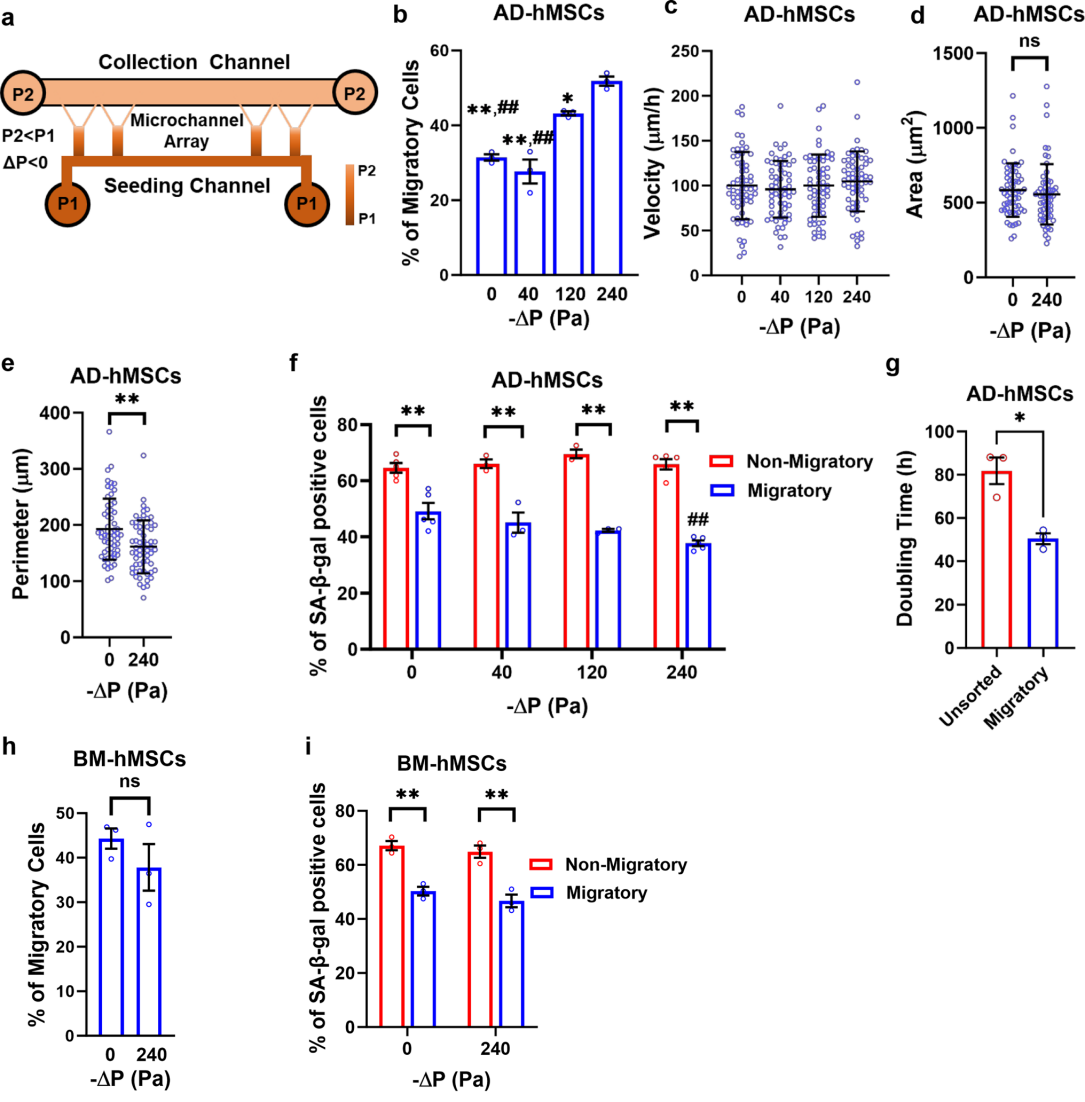
Pressure-driven flow improves MAqCI's capability to remove senescent adipose-derived hMSCs. **a** Schematic representation of a pressure gradient in the symmetric device. **b** Percentage of migratory adipose-derived hMSCs at passage 6 following exposure to different ΔP. At least 88 cells analyzed per experiment; 3 independent experiments; *p < 0.05, **p < 0.01 relative to ΔP = − 240 Pa and ^##^p < 0.01 relative to ΔP = − 120 Pa. **c** Velocity of migratory adipose-derived hMSCs at passage 6 following exposure to different ΔP. n = 60 cells; Data pooled from 3 independent experiments. **d** Area of migratory adipose-derived hMSCs at passage 6 following exposure to ΔP = 0 and ΔP = − 240 Pa. n = 60 cells; Data pooled from 3 independent experiments. **e** Perimeter of migratory adipose-derived hMSCs at passage 6 following exposure to ΔP = 0 and ΔP = − 240 Pa. n = 60 cells; Data pooled from 3 independent experiments; **p < 0.01. **f** Percentage of migratory and non-migratory adipose-derived hMSCs stained positive for SA-β-Gal at passage 6 following exposure to different ΔP. At least 27 cells analyzed per experiment; 3 independent experiments; **p < 0.01, ^##^p < 0.01 relative to migratory ΔP = 0. **g** Doubling time of migratory and unsorted, passage 6 adipose-derived hMSCs isolated from the collection and seeding channels, respectively. Prior to isolation, hMSCs were subjected to a ΔP of − 240 Pa. 3 independent experiments; *p < 0.05 **h**. Percentage of migratory bone marrow-derived hMSCs (BM-hMSCs) at passage 6 following exposure to ΔP = 0 and ΔP = − 240 Pa. At least 106 cells analyzed per experiment; 3 independent experiments. **i** Percentage of migratory and non-migratory bone marrow-derived hMSCs stained positive for SA-β-Gal at passage 6 following exposure to ΔP = 0 and ΔP = − 240 Pa. At least 45 cells analyzed per experiment; 3 independent experiments; **p < 0.01. Values represent mean ± SD (**c**–**e**) or mean ± SEM (**b**, **f**–**i**).

Systemically administered hMSCs promote regeneration by homing to injured or inflammatory tissues [41, 42]. During this process, hMSCs respond to fluid forces, such as those arising from blood, transmural or interstitial flow. Prior work has demonstrated that these forces can stimulate MSC motility and trigger downstream migration [41, 43]. Thus, we investigated how fluid flow impacted the ability of MAqCI to detect highly motile, presenescent hMSCs. To generate a flow field, we applied negative pressure differentials (ΔP) ranging from 0 to − 240 Pa across symmetric Y-shaped microchannels. This was achieved by manipulating the height of the medium in the inlet relative to the outlet wells (Fig. 5a). Microbeads were used to validate the presence of fluid flow in the microchannels (suppl. Video 3). Our results revealed that the percentage of highly motile adipose-derived hMSCs scaled with –ΔP (Fig. 5b), while their velocity and area remained unchanged (Fig. 5c and d). Moreover, staining for integrin β1, a key integrin used by MSCs for binding to collagen type I [44], revealed that integrin β1 adhesions were comparable in both migratory and non-migratory cells under flow conditions (Suppl. Figure 1e and f), suggesting that integrin β1 is not responsible for the migratory differences between these two subpopulations.

Fluid flow triggered by ΔP of − 240 Pa decreased the perimeter of adipose-derived hMSCs and the proportion of migratory hMSCs stained positive for SA-β-Gal from ~ 50% at static conditions (ΔP = 0) to ~ 38% at ΔP of − 240 Pa (Fig. 5e and f). To further confirm that highly motile adipose-derived hMSCs exposed to fluid flow had a presenescent phenotype, we isolated these cells from the collection channel and compared their proliferation rate to that of cells collected from the seeding channel (Fig. 5a). The latter cell population contained both migratory and non-migratory cells and thus closely resembled the unsorted population. Our results showed that migratory hMSCs displayed a markedly lower doubling time relative to unsorted cells (Fig. 5g). Furthermore, we conducted experiments utilizing hMSCs sourced from a distinct anatomical site, namely, the bone marrow. Consistent with our results in adipose-derived hMSCs, we observed that migratory cells represented ~ 45% of passage 6 bone marrow-derived hMSCs (Fig. 5h) and displayed reduced levels of senescence relative to their non-migratory counterparts (Fig. 5i). Interestingly, application of a ΔP of − 240 Pa failed to enrich the highly motile bone marrow-derived hMSC subpopulation or to reduce the percentage of senescent cells within this population (Fig. 5h and i), suggesting that hMSCs isolated from distinct anatomical locations may respond differently to fluid flow.

## Discussion

Before reaching damaged tissues, hMSCs must navigate through mechanically challenging environments, including microvessels, narrow openings between endothelium, micropores, and confining channels [42]. The inability of cells and their nuclei to squeeze into confining spaces can compromise cell migration, consequently hindering tissue repair and regeneration [39, 45]. Prior work has shown that senescent hMSCs have increased size [10, 11] and reduced motility, flexibility and deformability [32–35]. Moreover, senescent MSCs diminish the function of neighboring healthy cells, including presenescent MSCs, fibroblasts, and hematopoietic stem cells [46–48]. Eradication of senescent cells is sufficient to trigger cell/tissue regeneration in vivo [49–51], suggesting that their selective removal from hMSC cultures may lead to stem cell rejuvenation. Supporting this notion, smaller SSEA-4^+^ cells, separated from highly senescent, elderly hMSCs, display enhanced proliferation and reduced levels of SA-β-Gal compared to the unsorted, aged population [47].

Common strategies to eliminate senescent hMSCs are marginally successful (e.g. senolytics [27]), or not clinically relevant (e.g., reprogramming [14, 22–25]). Additionally, microfluidic approaches that eliminate senescent cells based solely on their larger size may inadvertently disregard fully functional subpopulations of larger stem cells [4, 5, 52]. Herein, we hypothesized that MAqCI, which facilitates the detection of migratory cell subpopulations [37, 38], would effectively distinguish between presenescent and senescent hMSCs. MAqCI is superior to conventional transwell- or two-dimensional-migration assays since it recapitulates critical biochemical, biophysical, and topographical cues of the in vivo microenvironment [37, 38]. Consistent with our hypothesis, we found that highly motile fibroblasts or hMSCs identified by the device possess smaller size and elevated velocity and persistence while concurrently displaying increased proliferation potential and lower levels of senescence. These cells can squeeze into tightly confined environments, as shown using a symmetric design featuring narrow 3 μm-wide branch channels. Apart from cell size and deformability, many other cell-intrinsic processes may regulate the ability of cells to migrate through the Y-shaped microchannels, including focal adhesion assembly/disassembly, actin polymerization, myosin II contractility, nuclear stiffening and cell volume regulation. Prior work has established the key role of focal adhesions, actin cytoskeleton and myosin II in 2D migration [53, 54]. We and others have shown that nuclear rigidity and cell volume regulation contribute to confined cell migration [55–59]. Because Y-shaped microchannels impose varying degrees of confinement on cells, it is likely that migratory cells employ different migration mechanisms in the wider channels compared to the narrower branches. Uncovering these mechanisms and understanding how in vitro cell propagation affects the drivers of both confined and unconfined MSC migration could offer valuable insights for the development of interventions aimed at enhancing MSC homing.

We also found that migratory cells show lower levels of DNA damage, contrary to expectations based on previous studies associating migration through microfluidic constrictions with increased occurrence of nuclear envelope rupture-dependent DNA damage [60–62]. The absence of confinement-induced DNA damage in migratory cells could be explained by the tall and narrow dimensions (*H* = 10 μm and *W* = 3 μm) of the constricted channels employed in this study. As shown using microchannels with identical cross-sectional areas, the frequency of confinement-induced nuclear rupture is markedly decreased when cells move inside rectangular channels with narrow widths rather than small heights [60, 63]. Such differential responses to distinct microchannel geometries are attributed to dorsoventral (top-to-bottom) cell polarity [56].

It is worth noting that while the relative abundance of highly motile and deformable cells is elevated in early passages, it declines during in vitro expansion. Cell exposure to fluid flow increases the fraction of migratory, adipose-derived hMSCs, presumably by stimulating a larger pool of stem cells to move directionally from the seeding channel to the narrow branches. Increased fluid flow also enhances the efficacy of MAqCI in eliminating senescent adipose-derived hMSCs, likely because presenescent cells in the seeding channel are more responsive to fluid forces compared to senescent cells. In contrast to adipose-derived hMSCs, human bone marrow MSCs fail to increase their directional movement in response to the specific flow conditions employed in this study, resulting in an identical percentage of highly motile bone marrow MSCs under both static and conditions. Prior work has demonstrated that migratory responses to fluid flow vary based on cell type and culture conditions. While migration with the flow has been observed in C3H10T1/2 murine MSCs [43], endothelial cells [64] and sparse MDA-MB-231 3D cultures [65], movement against the flow has been reported with densely populated MDA-MB-231 breast cancer cells in 3D hydrogels [65] and with keratocytes [66], and certain immune cells [67–70] in 2D environments. The directional cell behavior under flow conditions is influenced by various factors, such as chemokines and their receptors [71], mechanosensitive elements [72], and interactions between integrins and their ligands [70]. Further investigations involving a larger pool of donors are essential to understand why bone marrow-derived hMSCs respond differently to fluid flow compared to adipose-derived hMSCs.

Using MAqCI, we can isolate ~ 100-150 highly motile passage 6 adipose-derived hMSCs under flow conditions. Considering that we routinely handle ~ 50 devices per day, the number of sorted MSCs can increase to ~ 5,000-7,500. While this cell number falls short of the hundreds of millions needed for cellular therapies, it is adequate for the detailed genetic, and functional characterization of this novel hMSC subpopulation. This characterization may involve performing RNA-seq to identify genes that are differentially expressed between migratory cells and the unsorted cell population. It could also include examining the long-term potential of highly motile cells and investigating their clonogenic, differentiation, immunosuppressive, and proangiogenic capabilities. Uncovering novel biomarkers for migratory cells may enable the sorting of millions of these cells directly from the total population using FACS. Moreover, employing methods to preserve the phenotype of isolated cells during expansion, such as culturing MSCs in 3D spheroids [73] or on ECM produced by young cells [74, 75] could facilitate the attainment of clinically relevant cell numbers.

In summary, MAqCI allows for detecting and isolating a more homogeneous and highly motile presenescent stem cell population distinguished by specific morphological traits. It is well established that only a tiny fraction of systemically delivered MSCs can reach injury sites [30]. This diminished homing capacity of hMSCs may stem from the accumulation of senescent cells during stem cell expansion or may be attributed to the low levels or lack of expression of several key adhesion molecules necessary for tethering/adhesion on the endothelium (e.g., selectin ligands, αLβ2) and for homing (e.g., CXCR4) [76, 77]. In vitro propagation further reduces the expression of several chemokine receptors [78]. Because the migratory capability of administered MSCs is linked to improved outcomes in patients suffering from acute myocardial infarction [31], isolating and delivering highly motile, presenescent stem cells, could offer promising prospects for improving clinical outcomes.

## Materials and Methods

### Cell Lines and Cell Culture

Human dermal fibroblasts (GM05565) and hMSCs (Adipose-derived and Bone marrow-derived) were purchased from the Coriell Institute for Medical Research and the American Type Culture Collection (ATCC), respectively. The adipose-derived hMSCs originated from a 52-year-old donor, while the bone marrow-derived hMSCs were obtained from a 26-year-old donor. Human dermal fibroblasts were cultured in Dulbecco's modified Eagle's medium (DMEM; Gibco) supplemented with 10% (v/v) fetal bovine serum (FBS; Bio-Techne) and 1% (v/v) penicillin/streptomycin (10,000 U/ml; Gibco). Adipose- and bone marrow-derived MSCs were cultured in Mesenchymal Stem Cell Basal Media for Adipose, Umbilical and Bone Marrow-derived MSCs (ATCC) supplemented with either Mesenchymal Stem Cell Growth Kit for adipose-derived MSCs or bone marrow-derived MSCs (ATCC). Dermal fibroblasts and MSCs were cultured in an incubator at 37 °C and 5% CO_2_ and passaged every 5 and 3 days, respectively. All the experiments were conducted using cells within one passage number of the stated passage number. We estimate that the passage 6 bone marrow MSCs and adipose-derived hMSCs used in this study have undergone ~ 17-19 population doublings. This estimate is based on published reports indicating that MSCs typically undergo 7-9 population doublings during initial isolation and expansion period [79], along with our observation that hMSCs average two population doublings per passage during the first six passages. All cell lines were tested negative for mycoplasma with PCR (forward primer: GGGAGCAAACAGGATTAGATACCCT, reverse primer: TGCACCATCTGTCACTCTGTTAACCTC).

### Photolithography and Microchannel Device Fabrication

Replica molding was used to make straight as well as symmetric and asymmetric polydimethylsiloxane (PDMS)-based microchannel devices. Multilayer photolithography was employed to fabricate replica molds. Briefly, SU-8, an epoxy-based photoresist, was applied onto the surface of a silicon wafer using a spin coater, achieving a targeted thickness of ~ 10 µm. Next, the wafer was exposed to UV light through a photomask that featured straight, symmetric or asymmetric patterns in order to crosslink the photoresist. After washing out the uncrosslinked SU-8, a second layer of the photoresist was deposited onto the wafer and crosslinked, resulting in an additional layer with a thickness of ~ 50 µm. PDMS-based devices were created by mixing base elastomer with the curing agent at a 10 to 1 ratio, then pouring the mixture over the silicon wafer and baking it at 85 °C for about 1 hour. Next, PDMS devices were taken out from the master mold, punched to form inlet and outlet holes and  bound to coverslips after plasma treatment. All devices were coated with rat tail collagen type I (Corning, 20 µg/ml) solution for 1 hour at 37 °C.

### Cell Seeding and Generation of a Pressure Gradient

Cells were resuspended in the appropriate culture media at a concentration of 3–4 x 10^6^ cells/mL. 20 µl of the cell suspension was used to seed cells in the seeding channel. The devices were then incubated at 37 °C and 5% CO_2_ for 10-15 minutes to facilitate cell adhesion to the coated surfaces. Following cell adhesion, an equal amount of fresh cell culture medium was added to the inlet and outlet wells. For experiments involving fluid flow, the cell culture media height in the inlet relative to the outlet wells was adjusted, thereby creating a ΔP between the seeding and collection 2D-like channel. ΔP was calculated using the expression ΔP = ρgΔh, where ρ is the density of the culture media (1,000 kg/m^3^), g represents the gravitational acceleration (9.81 m/s^2^) and Δh is the culture media height difference between the inlet and outlet wells (Table 1). To ensure a consistent pressure gradient throughout the 16-hour experiment, the medium inside the devices was replaced after 8 hours with a new medium, adjusted to the prescribed height (Table 1).

**Table 1:** The vertical variation in height and the corresponding pressure differentials

~ Δh (cm)	0.41	1.22	2.45
ΔP(Pa)	40	120	240

### Quantification of the Percentage of Migratory Cells, Migration Velocity, Persistence, and Morphological Features

Images of devices containing straight or Y-shaped channels were recorded every 30 minutes for 16 hours using a Nikon Eclipse Ti2 Inverted microscope. The percentage of migratory cells was determined by dividing the number of cells that entered the narrower branch channels with the total number of cells that entered the feeder channel. The percentage of cells that exited straight microchannels was determined by dividing the number of cells that exited the straight channels with the total number of cells that entered these channels. Cell migration tracking was conducted using MTrackJ, and cell velocity and persistence were calculated using a custom-made MATLAB script. Migration velocity was defined as the net cell displacement divided by time, and migration persistence as net cell displacement divided by the total distance traveled. For morphological analysis, ImageJ's polygon area selection tool was employed to draw a closed region of interest (ROI) around the cell periphery. The measure ROI tool was then used to calculate parameters such as cell area, perimeter, aspect ratio, and solidity.

### Cell Tracking Using a Fluorescent Probe and Analysis of Mixed Cell Populations

To test MAqCI's capability of enriching presenescent cells, passage 15 fibroblasts were labeled with the CytoTrace™ Red CMTPX Dye (AAT Bioquest) in accordance with the manufacturer's staining protocol and then mixed with passage 28 fibroblasts at 1:3 ratio. The mixture of cells was then introduced into the microchannel devices. Bright-field and TRITC images were captured using a Nikon Eclipse Ti2 Inverted microscope over a period of 16 hours to monitor the migration of both unlabeled and labeled cells. The percentage of passage 15 cells in the seeding channel was quantified and compared with the percentage of passage 15 cells in the migratory cell population. As a control, migration experiments were carried out using labeled and unlabeled passage 15 fibroblasts to ensure that the dye had no impact on the fraction of highly motile cells.

### Immunocytochemistry and Quantification of Ki-67, Phospho-H2A.X Levels and Integrin β1

Following migration experiments, cells were fixed using 4% paraformaldehyde (Thermo Scientific) for 10-12 minutes at room temperature, permeabilized via 0.1% Triton X-100 (Sigma) for 15 minutes and blocked for 1 hour at room temperature using 1% BSA (Sigma), 2% goat serum (Vector Laboratories), and 0.01% Triton X-100. Next, cells were incubated with anti-Ki67 (clone 8D5, 1:400, Cell Signaling Technology), anti-phospho-histone H2A.X (Ser139, clone 20E3, 1:400, Cell Signaling Technology), and anti-Integrin beta 1 (Mouse monoclonal [12G10], 1:200, Abcam) antibodies at 4 °C overnight. The next day, samples were incubated for 2 hours at room temperature with Alexa Flour 488 goat anti-rabbit (1:200, Invitrogen) or Alexa Flour 568 goat anti-mouse (1:200, Invitrogen). Hoechst 33342 (1:2000, Biotium) was used to stain the nucleus. Negative control samples were not treated with primary antibodies.

The percentage of Ki-67 positive cells was quantified by dividing the number of cells expressing Ki-67 with the total number of cells in the corresponding cell population. DNA damage was assessed by measuring the total number of foci in the nucleus stained positive for phospho-H2A.X. The number of integrin β1 adhesions per cell and the total area of integrin β1 adhesions per cell were quantified using different plugins in ImageJ software [80]. Briefly, the fluorescent images underwent different processing steps aimed at subtracting and minimizing background noise, enhancing local contrast, and converting grayscale images to binary images. The Analyze Particles plugin was utilized to detect and outline individual integrin β1 adhesions, allowing their count and measure of their area.

### SA-β-Gal Staining and Quantifications

Senescence-associated beta-galactosidase (SA-β-Gal) activity in migratory and non-migratory populations was quantified using the Senescence β-Galactosidase Staining Kit (Cell Signaling Technology). Cells were fixed with 1X fixative solution for 10-15 minutes at room temperature and subsequently stained with the staining solution adjusted to a final pH of 6. The samples were sealed with parafilm to prevent evaporation and then incubated overnight at 37 °C in a dry incubator. Imaging was performed using the high-resolution Nikon DS-Fi3 Color Microscopy Camera.

### Imaging of Microbeads

To assess fluid flow within the microchannels, Fluoro-Max Dyed Green Aqueous Fluorescent Particles (Thermo Scientific- Microgenics Corporation) with the size of 1 μm were utilized. These microbeads were then mixed with cell culture media at a concentration of 10% v/v. Prescribed volumes of the mixture were introduced into the inlet and outlet wells to generate the desired ΔP across the Y-shaped microchannels. A Prime BSI Express high-speed camera capable of fast imaging with the rate of 20 frames per second mounted on a Nikon Eclipse Ti2 Inverted microscope was employed to image the flowing microbeads.

### Collection of Highly Migratory Cells and Analysis of Their Proliferation Capacity

Approximately 60,000 adipose-derived MSCs were introduced in the seeding channel of MAqCI devices as described above. Cells were allowed to migrate through the Y-shaped channels in the presence of ΔP = − 240 Pa. After 16 hours, migratory cells that entered the collection channel were washed with PBS and detached using Trypsin 0.25% (Cytiva). It was estimated that each device yielded ~ 100-150 migratory cells. To obtain a sufficient quantity of migratory cells for the proliferation assay, we pooled cells from 10 devices. Furthermore, we followed the same isolation protocol for collecting cells from the seeding channel. This cell population predominantly contained the initially seeded cells (60,000), as only a tiny fraction of these cells (~200) was positioned close enough to the feeder channel entrances to migrate through the Y-shaped channels. Consequently, the cells in the seeding channel consisted of both migratory and non-migratory cells, closely resembling the unsorted parental population. An approximately equal number of cells from both the collection and seeding channels were seeded into a 96-well plate. After 12 hours, cells were imaged and manually counted. Four days later, cells were fixed and stained with Hoechst 33342 (1:2000, Biotium). The Analyze Particle plugin in ImageJ software was used to count the individual cells. The doubling time for both the migratory and unsorted cells was calculated using the following formula: Doubling time (hours) =  [Culture time (hours) × (Ln2)] / [Ln (N_f_ / N_i_)], where N_f_ and N_i_ are the final and initial number of cells.

### Statistical Analysis

Each experiment was performed at least three times, unless stated otherwise. To discern statistical differences, student's t-test, one-way ANOVA test followed by Tukey's test for multiple comparisons, two-way ANOVA test followed by Tukey's test for multiple comparisons, or 3-way ANOVA followed by a Sidak's test for multiple comparisons were used. GraphPad Prism (v. 9 and 10) was used to perform the aforementioned statistical analysis.

## Supplementary Information

Below is the link to the electronic supplementary material.

**Suppl. Figure 1**. (a) Percentage of late passage fibroblasts that exit straight microchannels with a cross-sectional area of 100 μm^2^. 18 cells analyzed per experiment; 3 independent experiments. (b) A representative image showing SA-β-gal-positive adipose-derived hMSCs at passage 6. Scale bar, 200 μm. (c) Aspect ratio and (d) solidity of migratory and non-migratory fibroblasts in asymmetric or symmetric devices at passages 15 and 25. n≥ 52 cells; Data pooled from 3 independent experiments. (e) Number and (f) total area of integrin β1 adhesions in migratory and non-migratory adipose-derived hMSCs subjected to ΔP=-240 Pa. At least 10 cells analyzed per experiment; Data pooled from 3 independent experiments. Values represent mean ± SEM (a) or mean ± SD (b-f). (PPTX 498 kb)

**Suppl. Video 1**. A representative video showing a migratory fibroblast at passage 15 in the asymmetric device. (AVI 463 kb)

**Suppl. Video 2**. A representative video showing a non-migratory fibroblast at passage 15 in the asymmetric device. (AVI 627 kb)

**Suppl. Video 3**. A representative video showing microbeads flowing through the feeder channel and narrow branches in response to ΔP=-240 Pa. (MP4 2146 kb)

## Data Availability

The datasets generated in this study are available upon request.
