# Supplementary figures and images for: Leveraging Cell Migration Dynamics to Discriminate Between Senescent and Presenescent Human Mesenchymal Stem Cells
Source: Cell Mol Bioeng. 2024 Jul 20;17(5):385–99. doi: 10.1007/s12195-024-00807-0 (PMC11538215; doi:10.1007/s12195-024-00807-0)

## Slide 1
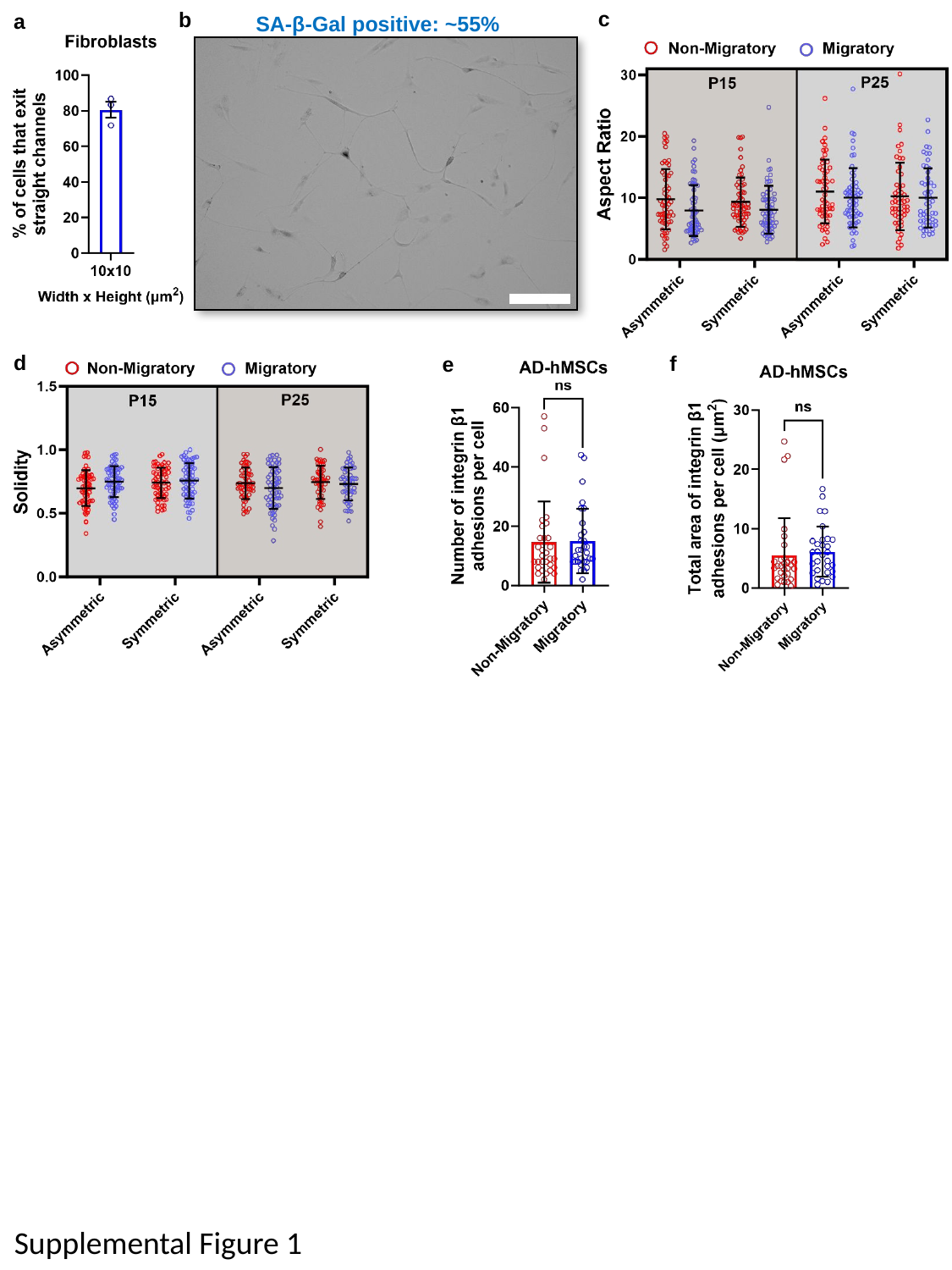

b
c
a
SA-β-Gal positive: ~55%
d
f
e
Supplemental Figure 1

Supplement: Supplementary file 1 — Suppl. Figure 1. (a) Percentage of late passage fibroblasts that exit straight microchannels with a cross-sectional area of 100 μm2. 18 cells analyzed per experiment; 3 independent experiments. (b) A representative image showing SA-β-gal-positive adipose-derived hMSCs at passage 6. Scale bar, 200 μm. (c) Aspect ratio and (d) solidity of migratory and non-migratory fibroblasts in asymmetric or symmetric devices at passages 15 and 25. n≥ 52 cells; Data pooled from 3 independent experiments. (e) Number and (f) total area of integrin β1 adhesions in migratory and non-migratory adipose-derived hMSCs subjected to ΔP=-240 Pa. At least 10 cells analyzed per experiment; Data pooled from 3 independent experiments. Values represent mean ± SEM (a) or mean ± SD (b-f). (PPTX 498 kb) [file 12195_2024_807_MOESM1_ESM.pptx]
